# The effects of pulsed electromagnetic field therapy on pain and physical functions in patients with soft tissue injuries: a systematic review of randomised controlled trials

**DOI:** 10.3389/fspor.2026.1694944

**Published:** 2026-02-05

**Authors:** Cheryl Shu Ming Chia, Sai-Chuen Fu, Violet Man-Chi Ko, Josephine Wing Hei Lai, Meng Zhou, Patrick Shu-Hang Yung, Samuel Ka-Kin Ling

**Affiliations:** 1 Department of Orthopaedics and Traumatology, Faculty of Medicine, The Chinese University of Hong Kong (CUHK), Hong Kong, Hong Kong SAR, China; 2 Office of Graduate Studies and Professional Learning, National Institute of Education, Nanyang Technological University, Singapore, Singapore

**Keywords:** Achilles tendon, ankle sprains, foot and ankle, pulsed electromagnetic field (PEMF), soft-tissue injuries/pathology, tendinopathy

## Abstract

**Background:**

Foot and ankle diseases are highly prevalent in both the general and athletic populations, frequently resulting in pain, impaired physical function, and a decreased quality of life. Pulsed Electromagnetic Field (PEMF) therapy has shown beneficial effects on pain by reducing inflammation and improving circulation, yet its efficacy in treating foot and ankle soft-tissue pathologies remains unclear. This systematic review aimed to evaluate the impact of PEMF therapy on pain and physical function among individuals with foot and ankle soft-tissue pathologies.

**Methods:**

A systematic literature search was conducted across Medline, Embase, Emcare (Ovid Nursing & Allied Health), Allied and Complementary Medicine Database (AMED), and Web of Science from database inception to May 15, 2025. Additional searches were performed using Google Scholar and clinical trial registries. Two reviewers independently screened studies and extracted data on pain and physical function outcomes.

**Results:**

Four randomised controlled trials (RCTs), comprising a total of 243 participants with a mean age of 48.79 years, were included in the review. In three of the four trials, PEMF therapy was administered alongside another conservative intervention, such as shockwave therapy, heel pads, or eccentric exercise, and compared to the conservative treatment alone. Only one study investigated the isolated effects of PEMF therapy vs. sham stimulation. Among the included studies, three reported statistically significant reductions in pain in the intervention groups compared to controls (*p* *<* *0.05*). However, only one of three studies demonstrated a significant improvement in physical function following PEMF therapy *(p* *<* *0.05*). Large heterogeneity in terms of treatment protocols and intervention parameters was observed across the studies which may limit the comparability of outcomes. No serious adverse events were reported; only minor skin redness was documented as a side effect.

**Conclusion:**

PEMF therapy appears safe and effective for reducing pain in individuals with various foot and ankle soft-tissue pathologies. However, the findings on the PEMF therapy in improving physical function remain inconclusive. Future research should focus on a large-scale, standardised setting, including the PEMF therapy protocol, to evaluate the efficacy of PEMF therapy on both pain and functional outcomes in this specific population.

**Systematic Review Registration:**

https://www.crd.york.ac.uk/PROSPERO/view/CRD420251076499, PROSPERO CRD420251076499.

## 1 Introduction

Foot and ankle injuries are among the most common and debilitating musculoskeletal conditions affecting both the general population and athletes (1, 2). According to Fong et al. (3), the ankle is the most frequently injured site in 24 out of 70 sports studied. This high incidence attributed to the region's complex biomechanics, where stability and mobility are maintained through a coordinated interplay between dynamic stabilisers (muscles, tendons) and static restraints (ligaments, joint capsule) (4, 5). These structures support weight-bearing, transmit forces between the lower limb and the ground, and enable efficient locomotion and posture control (6). Furthermore, the foot and ankle act as shock absorbers, adjusting to uneven surfaces and functioning as rigid levers during propulsion (6). Hence, disruption to this system can result in pain, instability and an increased risk of injuries such as ankle sprains, tendinopathy, bursitis, fractures, or dislocations, often leading to functional impairment and reduced quality of life (7–9).

Soft-tissue injuries of the foot and ankle are particularly prevalent. It is estimated that 30%–50% of all sports-related injuries involve soft tissue injuries (10), with incidence varying by sport. In the clinical setting, soft-tissue musculoskeletal injuries account for more than 50% of all musculoskeletal complaints in the United States annually (11, 12), representing a significant healthcare burden, with direct costs exceeding USD 15.8 billion annually (13). Therefore, appropriate treatment is essential to prevent long-term complications.

Soft tissue injuries can be broadly classified as acute or chronic. Previous evidence suggests that the common soft-tissue pathologies of the lower limb include ankle sprains, Achilles tendinopathy, and plantar fasciitis (14). Acute injuries such as ankle sprains typically result from sudden trauma, with LAS accounting for up to 77% of lower-limb soft-tissue injuries in the lower limb (15). These often occur due to the excessive supination of the rearfoot on an externally rotated lower limb during gait (16). During injury, the lateral ligament complex is stretched or torn, triggering an inflammatory response that can disrupt sensorimotor function and hinder healing (17). The long-term effects are significant: up to 40% of individuals with LAS will develop chronic ankle instability (CAI) (17). If left untreated, this may progress to osteochondral lesions of the talus (OLT) (18), early post-traumatic osteoarthritis and long-term joint dysfunction (19).

Chronic soft-tissue injuries, including Achilles tendinopathy (AT) and plantar fasciitis, are typically caused by overuse and repetitive microtrauma (20). AT affects up to 6% of the general population and as many as 50% of elite endurance runners (21), while plantar fasciitis is the most common cause of chronic heel pain, affecting up to 10% of the general population, particularly those who are physically active (22). Mechanical overloading without sufficient recovery may lead to persistent inflammation, maladaptive tissue remodelling, and chronic low-grade inflammation, contributing to tendon degeneration and pain (23). In plantar fasciitis, repetitive strain causes microtears in the plantar fascia, leading to stiffness and pain (24). This process is exacerbated by local inflammation and fibroblastic proliferation (25, 26). Calcaneal spurs frequently develop as a protective response to chronic traction of stress, forming a triangular bony protrusion at the bottom of the heel (27, 28). These injuries often develop insidiously and may remain undetected until they significantly impair function (29).

The current standard of care for soft-tissue foot and ankle injuries includes conservative treatment such as rehabilitation exercises, non-steroidal anti-inflammatory drugs (NSAIDs), corticosteroid injections, and biophysical modalities, including ultrasound therapy, neuromuscular electrical stimulation (NMES), and extracorporeal shockwave therapy (ESWT) (30–33). While these approaches may offer temporary relief of symptoms, the treatment effectiveness is not without limitations. For example, prolonged NSAIDs may cause gastrointestinal complications (34), and corticosteroid injections, though effective in the short term, have been associated with high recurrence rates and potential for tissue degeneration (35). Meanwhile, physical therapies can be categorised into thermal, mechanical, and electromagnetic (36). However, evidence on the effectiveness of these physical agents is unclear, and their use remains controversial (37–45).

Similarly, the exercise-based rehabilitation has its own limitations. For instance, although stretching protocols have been shown to alleviate symptoms in individuals with plantar fasciitis (31–33), up to 40% of patients continue to experience persistent symptoms even two years after diagnosis (32). Likewise, a review reported that exercise training alone may not consistently reduce pain in patients with AT (31). Common ankle-strength programmes using resistance bands may not mechanically train the key stabilising muscles (i.e., peroneal longus), which plays a crucial role in lateral ankle stability (46). Therefore, these findings highlight a critical gap in conventional rehabilitation, revealing the need for an innovative, evidence-based therapeutic approaches that promote deep tissue repair and long-lasting functional improvements.

Pulsed electromagnetic field (PEMF) therapy is a biophysical treatment that was first approved by the Food and Drug Administration (FDA) for treating bone non-unions in 1979 (47). Since then, clinical studies have consistently demonstrated that PEMF therapy accelerates wound healing, heals fractures, treats soft tissue injuries, and alleviates inflammation, making it a promising treatment for various musculoskeletal conditions, including lower back pain (48), fractures (49), and knee osteoarthritis (50). PEMF therapy delivers a specific, low-frequency electromagnetic field that generates bioelectric currents within tissues (51), thereby modulating cellular activity without producing heat or stimulating nociceptors (52, 53). Unlike surface electrical stimulation, PEMF therapy can penetrate deeper tissues without causing discomfort and modulate cellular processes, thereby targeting inflammatory responses and promoting regeneration in areas less accessible to conventional treatments (54–56). The energy is emitted as a sequence of impulses with very short pulse durations and a much longer “off” period than the “on” period, demonstrating that a lower dose is delivered to the patient and that any heat produced is dissipated by the circulation (57). Generally, PEMF therapy can mitigate the catabolic effects of systemic inflammation, particularly those mediated by interleukin-1β and TNF-α, while promoting tissue repair by upregulating vascular endothelial growth factors (58). Therefore, this unique mechanism may offer a distinct advantage to promote tissue regeneration in deep anatomical regions that are often challenging to target with topical or superficial treatments (55).

Despite growing interest in PEMF therapy, high-quality evidence supporting its application in soft-tissue foot and ankle injuries remained limited. To date, only one narrative review has reported on the effectiveness of PEMF therapy for bony structures related to foot and ankle injuries, including osteogenesis, pain relief, and joint preservation, among patients with bone marrow oedema, osteochondral defects, and fractures (59). However, this review did not systematically identify existing evidence or assess the effectiveness of PEMF therapy for various soft-tissue pathologies. To the best of our knowledge, no systematic review has evaluated the effectiveness of PEMF therapy for foot and ankle soft-tissue injuries using the results from randomised controlled trials (RCTs). Thus, this systematic review aims to assess the current evidence on the effectiveness of PEMF therapy, compared with control interventions, in improving pain and functional outcomes in patients with soft-tissue foot and ankle injuries. It is hypothesised that PEMF therapy, when used as an adjunct to standard care, will lead to greater improvements in clinical outcomes compared to sham or standard care alone.

## 2 Methods

The systematic review was conducted in accordance with the Preferred Reporting Items for Systematic reviews and Meta-Analyses (PRISMA) guidelines. The review protocol was pre-registered with PROSPERO (CRD420251076499).

### 2.1 Systematic literature search

The authors have searched various databases, including Medline, OVID Embase, OVID Emcare (Ovid Nursing & Allied Health), Allied and Complementary Medicine Database (AMED), and Web of Science. We also utilised Google Scholar and the clinical trial registry for additional publications. This database was last searched from its inception through May 2025. The following keywords and Boolean operators were used: “PEMF” OR “pulsed electromagnetic fields” OR “pulsed electromagnetic field therapy” OR “Diapulse” OR “puls* electromagnetic* field* therap*” AND “ankle instability” OR “recurrent ankle sprain” OR “chronic ankle instability” OR “chronic lateral ankle instability” OR “CAI” OR “CLAI” OR “functional ankle instability” OR “ankle injur*” OR “ankle sprains” OR “plantar heel pain,” OR “plantar fasciitis,” OR “Achilles Tendon disease” OR “chronic Achilles Tendinopathy” OR “Achilles Tendinosis” OR “Ankle Joint injur*” OR “soft tissue injur*” OR “Foot injur*” OR “Foot and ankle injur*” in the English literature.

### 2.2 Inclusion and exclusion criteria

The inclusion criteria for the studies included in this review followed the Patient/population, Intervention, Comparison, Outcome (PICO) model:

**Population:** Adult patients (>18 years old) who were clinically diagnosed with foot and ankle pathologies (including soft tissues and fractures).

**Intervention:** Studies reporting on the effects of PEMF therapy.

**Control:** Studies that have included a control group of sham-PEMF therapy only or combined with other conventional physiotherapy.

**Outcomes:** Studies reporting on the clinical outcomes of PEMF therapy on clinical outcomes such as pain, physical and balance functions, foot and ankle functions or muscle health.

#### 2.2.1 Study design: randomised controlled trials

Studies were excluded if they were presented only as an abstract or poster, were animal studies, were non-English papers, or focused on the pediatric population. The outcomes were excluded if they did not measure pain or function using validated instruments. The information extracted included the author's last name, year of publication, study design, sample size, treatment technique, number of arms, frequency and duration of treatment, outcomes, and conclusion.

#### 2.2.2 Study selection

Two independent reviewers (CSMC and KVMC) screened both titles and abstracts from the various databases. The two reviewers independently read the selected articles and applied the eligibility criteria to the full-text reports. Disagreements were either resolved through consensus or by a third reviewer (JW) if necessary (60).

#### 2.2.3 Data extraction

Data were systematically extracted and organised into a comprehensive table from the included studies. A narrative synthesis was performed to summarise the findings. Extracted information included the following categories: authors' names, year of publication, study design, patient characteristics, diagnosis, intervention and control groups, treatment regimen, frequency, intensity, follow-up time points, assessments, and results. The results were presented with corresponding *p-values*. Due to the limited availability and heterogeneity of the data, a meta-synthesis was not feasible and therefore was not conducted. However, the review included a qualitative synthesis focusing on pain and physical functional outcomes to assess the extent of improvement following PEMF therapy. A forest plot without the pooled outcome was conducted to show the direction and magnitude of effect across studies for the pain outcome. A forest plot with a pooled estimate was not generated for physical function outcomes due to heterogeneity in outcome measures and inconsistent effect directions across studies.

### 2.3 Methodological quality assessment

The reviewers assessed the risk of bias in the studies considered using the PEDro scale. Both authors agreed upon the consensus regarding the PEDro scale for each article. This scale is presented objectively with detailed scores of 11 items with a total score ranging from 0 to 10 (item 1 is not scored), and each item is scored “yes” (1 point) or “no” (0 point). In addition, scores above 6 are considered high quality, and scores below 6 indicate greater potential for bias to affect the trial.

### 2.4 Rating quality of evidence

The Cochrane Grading of Recommendations, Assessment, Development and Evaluation (GRADE) system was used to evaluate the level of evidence of each outcome. The assessment was conducted in alignment with the GRADE guidelines. The level of evidence was divided into high, moderate, low and very low (61).

## 3 Results

### 3.1 Study selection and characteristics

A total of 62 articles were found in the databases; articles were removed due to duplicates (*n* = 23), and were irrelevant (*n* = 25).

**Figure 1:**
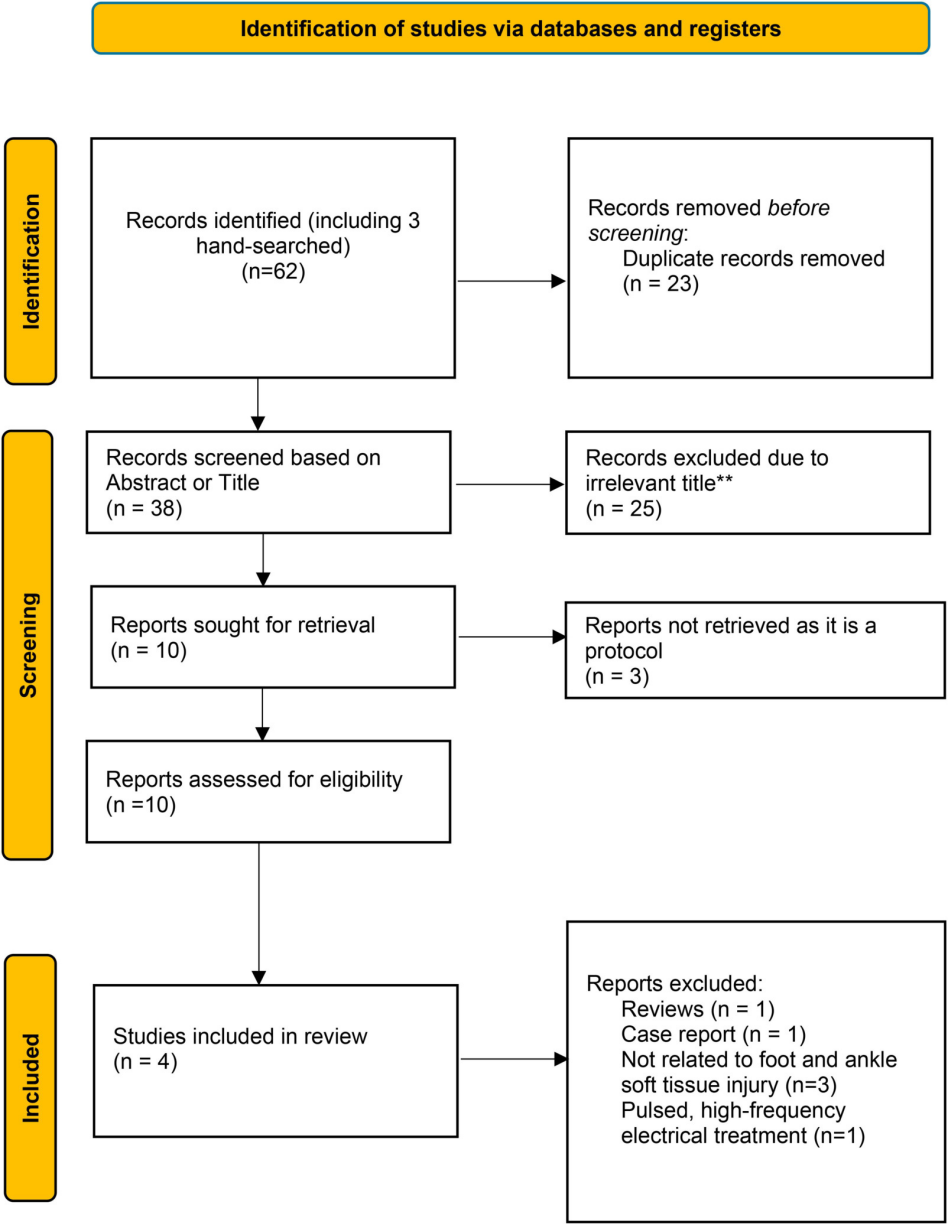
Preferred reporting items for systematic reviews and meta-analyses (PRISMA) flow diagram of the eligibility process follows PRISMA 2020. RCT, randomised controlled trial (105).

Following the exclusion of case reports (*n* = 1), review articles (*n* = 1), studies unrelated to soft-tissue injuries (*n* = 3), and those employing pulsed high-frequency electrical stimulation (*n* = 1), a total of four studies met the inclusion criteria for this systematic review (Figure 1). Of those selected articles, one study investigated the effects of PEMF therapy on acute ankle sprain (62), two studies focused on Achilles tendinopathy (63, 64), and one study examined calcaneal spurs, a condition that is associated with plantar fasciitis (65).

Overall, the studies included 243 participants (98 females and 42 males), with a mean age of 48.79 years. However, two studies, Pennington et al. (62) and Gerdesmeyer et al*.* (63), did not report participant demographic data such as age and gender (Table 1), and one study, Ozturk et al. (65) reported a median age of 51 years old. Due to significant heterogeneity in the outcome measures used to assess clinical endpoints such as pain and physical function, a quantitative synthesis through meta-analysis was not appropriate as data were too sparse due to the small number of trials. One study evaluated PEMF therapy against a placebo-controlled PEMF therapy. One study assessed the combined effects of PEMF therapy and heel cushioning against heel cushioning alone, and another compared PEMF therapy combined with extracorporeal shockwave therapy against shockwave therapy alone. There was a variation in the devices and application protocols used for PEMF therapy. The frequency ranged from 3 Hz to 100 Hz, and field intensity ranged from 1 millitesla (mT) to 80 mT, with one study which did not specify the parameters for the PEMF therapy (62). In addition, the treatment regime was not standardised, with intervention durations ranging from a single day to 8 weeks. The frequency of application was between 1 and 5 sessions per week, with each session lasting from 10 to 60 min. Follow-up periods also differed, ranging from immediate post-intervention assessments to 12 weeks after the intervention.

**Table 1:** Characteristics and results from included studies for pain.

Name of Authors and year of publication	Type of study design	Characteristics of patients	Diagnosis	Intervention and Control Groups	Treatment regimen	Frequency	Intensity	Follow-up timepoint	Assessment	Results
Pennington et al*.* (1993) (62)	RCT	*n* = 50	Ankle sprain (Type 1 and Type 2)	Intervention group: Diapulse therapy (*n* = 25)	1 h for 1 session	High frequency	Did not specify	Post treatment	Tank water displacement for swelling VAS for pain	Significant reduction of edema 4 times in intervention group compared to control group *(p* *<* *0.05).* Twice as many people in diapulse group reduced in pain compared to control group (*p* < 0.05)
		Gender M/F: not specified		Control group: Placebo (no diapulse therapy) (*n* = 25)		0Hz	0mT			
		Mean age: not specified								
Gerdesmeyer et al. *(*2017) (63)	Parallel study	*n* = 53	Mid portion Achilles tendinopathy	Intervention group: PEMF therapy and heel cushion (*n* = 28)	20 min/ twice per week/4 weeks	3Hz	80 mT	12 weeks follow-up	VAS pain score	VAS pain scores significantly decreased in the PEMF group compared to control group (*p* < 0.05)
		Gender Not specified		Control group: Heel cushion only (*n* = 25)	–	–	–			
		Mean age: 44.7 (9.1) years								
Ozturk et al.*,* (2023) (65)	RCT	*n* = 75	Calcaneal spurs	Intervention group: PEMF therapy and shockwave therapy (*n* = 40)	20 min/5 sessions at 4 days interval	PEMF therapy = 10−100Hz	2mT	Post treatment, 3rd month follow-up	VAS scores	PEMF therapy + shockwave therapy significantly improved VAS pain score compared to only shockwave therapy
		Gender M: 9 F: 66		Control group: Only Shockwave therapy (*n* = 35)	5 sessions at 4 days intervals	(Roland Health, Elettronica Pagani) (6.0 Hz, 500 shock waves, 1.7 bar pressure)				
		Median age = 51 years								
Ko et al. (2024) (64)	RCT	*n* = 65	Achilles tendinopathy	Intervention group: PEMF therapy + Eccentric Exercise	10 min/twice per week/8 weeks	50Hz	1 mT	4th weeks, 8th weeks and 12th weeks	NPRS-sports	No significant between-group difference in pain, between intervention and control group (*p* > 0.05). Significant within group differences was found in the NPRS-pain score for sport between both PEMF and sham group (*p* < 0.05)
		Gender M: 33 F: 32		Control group: Sham PEMF therapy and eccentric exercise	10 min/twice per week/8 weeks	0 Hz	0 mT			
		Mean age: 52.87 years								

VAS, visual analog scale; NPRS, numerical pain rating score; SD, standard deviation.

### 3.2 Study findings

#### 3.2.1 Pain symptoms

**Figure 2:**
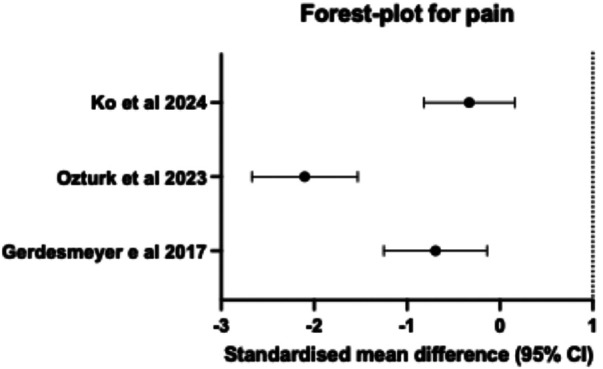
Forest plot of the pain intensity in the different foot and ankle soft tissue pathologies.

All included studies reported pain outcomes using either the visual analogue scale (VAS) or the numerical pain rating scale (NPRS) (Table 1). Among these, two of the four studies, Gerdesmeyer et al. (63) and Ozturk et al. (65), reported significant pain reduction with PEMF therapy compared to the control group at all time points *(p* *<* *0.05).* In contrast, Ko et al. (64) reported no significant difference in pain reduction between the intervention and control groups. The standardised mean difference (SMD) (95% confidence interval) presented by Ozturk et al. (65) was the highest, with a SMD (95% CI) of −2.10 (−2.67, −1.53), followed by Gerdesmeyer et al. (63) with a SMD (95% CI) of −0.69 (−1.25, −0.14), as observed in the forest plot (Figure 2). These RCTs demonstrated a positive impact on pain management across various foot and ankle soft-tissue pathologies, except for the study by Ko et al. (64), which reported the lowest SMD (95% CI) of −0.33 (−0.82, 0.16). This concurs with the findings from the meta-synthesis in Table 2, where Ozturk et al. (62) reported the highest significant reduction of pain score in the intervention group, with a low mean score of 1 point compared to the control group, with a mean score of 7 points. This is followed by Gerdesmeyer et al. (63), who showed that the intervention group had a significantly lower pain score of 3.61 compared to the control group with a mean score of 4.88, *p* *<* *0.05.* The changes in pain scores could not be determined in Pennington et al. (62) because no NPRS scores were reported for the pre- and post-intervention periods. Ko et al. (64) reported that both the control and intervention group did not demonstrate any between-group differences in NPRS pain scores (*F* = 1.345; *P* = 0.253).

**Table 2:** Qualitative synthesis of the PEMF therapy effects on pain in soft tissue injuries.

Reference	Scale	Improvement between control and experimental
Calcaneal Spurs		
Ozturk et al. (2023) (65)	VAS pain score at rest	The pain score was significantly reduced in the intervention group (ESWT and PEMF) with a median score of 1 (1) compared to the control group (ESWT) with a median score of 7 (4), *p* *<* *0.001*
Achilles Tendinopathy		
Gerdesmeyer et al. (2017) (63)	VAS pain score at rest	The mean pain score was significantly reduced in the intervention group [3.61 (SD 2.01)] compared to the control group [4.88 (1.60)], *p* *<* *0.05*
Ko et al. (2024) (64)	NPRS at rest	No significant improvement in pain score was found between the intervention and control group *(p* *>* *0.05).* The pain score was reduced to 3.60 in the intervention group versus 4.30 in the control group, with (*F* = 1.345; *P* = .253)
Lateral ankle sprain		
Pennington et al. (1993) (62)	NPRS pain score at rest	A higher % of participants (64%) in the intervention group indicated alleviation in pain compared to the control group (33.33%). No NPRS score was reported

#### 3.2.2 Physical function

Physical functions were evaluated in three of the four studies using different outcome tools. Ko et al. (64), Gerdesmeyer et al (63), and Ozturk et al. (65) employed the Victorian Institute of Sport Assessment-Achilles (VISA-A) (66), the Role-Maudsley (65) and the Foot Function Index (FFI) (67), respectively. Both Gerdesmeyer et al. (63) and Ko et al. (64) did not show a significant between-group difference in PEMF vs. the control group, despite an improvement in physical function were observed in both groups, respectively. Only Ozturk et al. (65) reported that PEMF therapy significantly improved physical function compared with the control group (Table 3). A meta-synthesis was conducted to summarise the results on physical functions. In the meta-synthesis (Table 3), Ozturk et al. (65) showed the highest change in the between-group mean difference, with a difference of 35 points between the two groups (Table 4).

**Table 3:** Characteristics and main results From included studies for foot and ankle physical functions.

Name of Authors and year of publication	Type of study design	Characteristics of the patients	Diagnosis	Intervention and Control Groups	Treatment regimen	Frequency	Intensity	Measured timepoint	Assessment	Results
Gerdesmeyer et al. (2017) (63)	Parallel study	*n* = 53	Mid portion Achilles tendinopathy	**I**ntervention group: PEMF therapy + heel cushion (*n* = 28)	20 min/twice per week/4 weeks	3 Hz	80 mT	12 weeks follow-up	Role-Maudsley score	Both intervention and control group significantly improved the role-maudsley scores (*p* < 0.05). No significant between group differences in both the intervention and control group
		Gender: not specified		Control group: Heel cushion only (*n* = 25)	-	-	-			
		Mean age: 44.7 (9.1) years old								
Ozturk et al. (2023) (65)	RCT	*n* = 75	Calcaneal spurs	Intervention group: PEMF therapy and shockwave therapy	20 min/5sessions/4 days	Low frequency PEMF therapy = 10-100Hz	20G (PEMF)	Immediate post treatment, 3rd month follow-up	Foot Function Index (FFI)	PEMF therapy and shockwave therapy significantly improved FFI pain, disability, activity limitation compared to only shockwave therapy, (*p* < 0.05)
		Gender: M:9 F:66		Control group: Shockwave therapy		Shockwave therapy = 6.0 Hz, 500 shockwaves, 1.7 bar pressure	–			
		Mean Age: 51 years old (8.25)								
Ko et al. (2024) (64)	RCT	*n* = 65	Achilles tendinopathy	Intervention group: PEMF therapy + Eccentric Exercise	10 min/twice per week/8 weeks	50Hz	1mT	4th weeks, 8th weeks and 12th weeks	Victorian-Institute of Sport Assessment-Achilles (VISA-A)	No significant between-group difference in self-reported functional outcomes between intervention and control group (*p* > 0.05) Significant within group differences were found in both PEMF and sham group for the VISA A score (*p* < 0.05)
		Gender: F: 32 M: 33		Control group: Sham PEMF therapy + eccentric exercise	10 min/twice per week/8 weeks	0Hz	0Mt -			
		Mean age: 52.87								

**Table 4:** Qualitative synthesis of the PEMF therapy effects on physical function in soft tissue injuries.

Reference	Scale	Improvement between control and experimental
Calcaneal Spurs		
Ozturk et al. (2023) (65)	Foot Function Index (Functional activity limitation) A higher score indicates a worse foot function	The experimental group showed a superior clinical outcome with an average of 35 points lower than the control group at final follow-up (*p* *<* *0.001)*. Mean score of FFI for experiment group is 5 (5.75) while for the control group is 40 (27)
Achilles Tendinopathy		
Gerdesmeyer et al. (2017) (63)	Role-Maudsley A lower score indicates better improvement	No significant improvement between the two groups
Ko et al. (2024) (64)	Victorian-Institute of Sport Assessment-Achilles (VISA-A) Higher score indicates less pain and better physical functions	No significant improvement between the two groups

### 3.3 Quality assessment

The mean PEDro score to evaluate the quality of the included studies was 7.75 (range 5–10). The results indicated that four studies (62, 64, 65, 68) had excellent quality, one study (48) had good quality (69), and another (42) had fair quality (63). The three studies did not report blinding of patients, the therapist who administered the intervention, or the assessor who conducted the assessment (Table 5).

**Table 5:** Methodological quality assessment by PEDro.

Criteria for PEDRO scale ^a^													
Study	1	2	3	4	5	6	7	8	9	10	11	Total score	Quality
Pennington et al. (1994) ( 62 )	✓	✓	✓	✗	✓	✓	✓	✓	✓	✓	✓	9/10	Excellent
Gerdesmeyer et al. (2017) ( 63 )	✓	✗	✗	✓	✗	✗	✗	✓	✗	✓	✓	5/10	Fair
Öztürk et al. (2023) ( 65 )	✓	✓	✓	✓	✗	✗	✗	✓	✓	✓	✓	7/10	Excellent
Ko et al. (2024) ( 64 )	✓	✓	✓	✓	✓	✓	✓	✓	✓	✓	✓	10/10	Excellent

^a^ (1) Eligibility criteria were specified; (2) subjects were randomly allocated to groups; (3) allocation was concealed; (4) the groups were similar at baseline regarding the most important prognostic indicators; (5) there was blinding of all subjects; (6) there was blinding of all therapists who administered the therapy; (7) there was blinding of all assessors who measured at least one key outcome; (8) measures of at least one key outcome were obtained from more than one of the subjects initially allocated to groups; (9) all subjects for whom outcome measures were available received the treatment or control condition as allocated or, where this was not the case, data for at least one key outcome was analysed by “intention to treat”; (10) the results of between-group statistical comparisons are reported for at least one key outcome; (11) the study provides both point measures and measures of variability for at least one key outcome.

For each measurement outcome, the GRADE system was used to analyse the level of evidence. The evidence quality for pain and physical function was rated as low (Table 6). The systematic review may not be entirely free of publication bias, as we included only published trial reports. Furthermore, insufficient reporting of trial methods may hinder the assessment of bias within this review. One of the clinical trials did not specify an adequate procedure for treatment allocation (63).

**Table 6:** Evidence quality rated using the GRADE approach.

Outcomes	No. of studies	Sample Size	Risk of Bias	Inconsistency	Indirectness	Imprecision	Publication Bias	Evidence Quality
Pain	4	243	Some concerns	Serious	Not serious	Not serious	Detected	Low
Physical Functions	3	193	Some concerns	Serious	Not serious	Not serious	Detected	Low

## 4 Discussion

This systematic review synthesised and evaluated the evidence on PEMF therapy for various foot and ankle soft-tissue injuries. Across the four studies reviewed, three showed promising results but with low-to-moderate certainty evidence and considerable heterogeneity regarding the improvement in short-term pain following PEMF therapy. Conversely, the effects of PEMF therapy on self-reported physical functioning remain inconclusive, as only one of the three studies reported a significant improvement in physical function after PEMF therapy.

The PEDro scale was used to evaluate the quality of the included studies (Table 5). Three included studies were RCTs and scored more than 7 out of 10 points on the PEDro scale (62, 64, 65). One study was scored 5 out of 10 points on the PEDro scale (63), as it did not adequately report how the randomisation was performed and the procedure to blind both assessors and participants. Three trials did not provide sufficient information to accurately assess the concealment of treatment allocation (62, 63, 65). Therefore, the most frequently considered high risk was the lack of blinding of participants and assessors.

The overall certainty of evidence using the GRADE approach (Table 6) rates the evidence level for both pain and functional outcomes as low, primarily due to inconsistency arising from the different types of soft-tissue pathologies and disease stages (acute vs. chronic). The inclusion of both chronic and acute soft-tissue pathologies may affect treatment response. Regarding the certainty of the evidence, 25% of the studies did not blind the assessor and therapist who administered the therapy (63). Therefore, the overall findings regarding treatment effectiveness should be evaluated with caution.

In the current systematic review, Pennington et al. (62) were among the first to demonstrate that high-frequency PEMF therapy could reduce pain in individuals with acute ankle sprains, as it was observed that twice as many patients allocated to the intervention (16/25) group reported a reduction in pain compared to the control group (8/25). The study employed a high-frequency PEMF therapy (Diapulse) that was postulated to increase collagen formation and accelerate healing in ligament/tendon injury (62). Furthermore, the results showed that PEMF therapy reduced swelling by 4-fold in the intervention group compared to the control group, indicating that PEMF therapy can ameliorate the inflammatory process while stimulating the release of anti-inflammatory cytokines (e.g., interleukin 10) (70). The findings corroborated those of previous studies using high-frequency PEMF therapy to reduce pain in ankle sprains (71), rotator cuff tendinopathy (72), and shoulder impingement syndrome (73), suggesting that high-frequency PEMF therapy may alleviate pain symptoms. However, the study on the effectiveness of PEMF therapy and ankle sprain was excluded from this systematic review due to restricted access to the full article (71).

Additionally, Ozturk et al. (65) reported that combining a moderate-intensity and low-frequency PEMF therapy alongside with ESWT may elicit a synergistic effect in reducing pain symptoms among patients with calcaneal spurs (65). This contrasts with the individual effects of ESWT and PEMF therapy, which did not show any superior outcomes over each other, as shown in a previous study (74). Essentially, PEMF therapy delivers electromagnetic energy to soft tissues, promoting bone healing, collagen synthesis, reducing inflammation, and tissue repair (75–77). It also enhances Ca2 + binding to calmodulin, thereby triggering nitric oxide release and stimulating growth factor secretion, which supports cartilage repair by modulating chondrocyte activity (78). Meanwhile, ESWT demonstrated a success rate of 50% to 94% in treating patients with plantar fasciitis. This is achieved through the use of high-energy acoustic waves, which effectively boost fibrotic activity, stimulate collagen production, initiate angiogenesis, facilitate subchondral bone remodelling, reduce inflammation, and promote tissue regeneration responses at both cellular and molecular levels (79, 80). Therefore, it is postulated that combining ESWT and PEMF therapy will accelerate the recovery mechanism and require a shorter treatment duration (20 min per session, five sessions in total, with a four-day interval) than other conventional treatments for decreasing inflammation and accelerating soft tissue healing (81). The findings of our systematic review corroborated the evidence from other similar studies, with one case-series study showing that 12 weeks of PEMF therapy could stimulate healing and improve function among individuals with plantar fasciitis (82). Similarly, another study using a similar biophysical therapy showed that pulsed radiofrequency electromagnetic field therapy demonstrated not only a 40% reduction in pain scores but also decreased pain medication use among individuals with plantar fasciitis compared to the control group (83). The findings from this systematic review further reinforced the conclusions of Mazzotti et al (59), suggesting that PEMF therapy can stimulate a strong anti-inflammatory mechanism and chondroprotective effect. Specifically, PEMF therapy can inhibit the release of pro-inflammatory cytokines interleukin-6 (IL-6), and interleukin-8 (IL-8) while upregulating anti-inflammatory mediators like IL-10. Additionally, PEMF therapy can increase proteoglycan synthesis and chondrocyte proliferation, in concert with insulin-like growth factor-1 present in both synovial fluid and articular cartilage, which play a key role in anabolic growth factors for cartilage metabolism recovery.

Regarding the effectiveness of PEMF therapy in individuals with AT, Gerdesmeyer et al. (63) showed that a high-intensity, low-frequency PEMF protocol, characterised by a frequency of 3 Hz and an amplitude of 80 mT, can stimulate mesenchymal stem cell activity and promote tissue repair (84). This is supported by a previous meta-analysis, which found that high-intensity PEMF therapy was associated with meaningful analgesic and regenerative effects in tendinopathic conditions without adverse effects (73). The proposed mechanism in pain alleviation by PEMF therapy includes the increase in intracellular calcium ions through cellular signaling modifications (85). Intracellular calcium ions bind to calmodulin, inducing nitric oxide production that diffuses to nearby smooth muscle cells, causing relaxation and vasodilation, while limiting the inflammation (85). These effects are not restricted to articular chondrocytes but may extend to other cell types, influencing pain perception, reducing oedema, and enhancing angiogenesis (85). Furthermore, PEMF therapy has been shown to increase oxygen delivery from red blood cells, thereby improving tissue oxygenation and enhancing local blood flow through vasodilation (86). This process is accompanied by alterations in blood ions, facilitating the removal of inflammatory mediators and metabolic waste from the affected area (86). Simultaneously, it promotes the influx of essential nutrients and endorphins, contributing to pain relief (86).

In contrast, a previous systematic review suggests that low-intensity PEMF therapy may not sufficiently activate the cellular signalling pathways required for effective tissue regeneration in tendinopathy, compared with high-intensity PEMF therapy (73, 87–89). This concurs with the findings of Ko et al. (64), showing that PEMF therapy does not elicit any superior treatment in improving pain symptoms compared with the control group (eccentric exercise and sham PEMF therapy). This may be possible as the PEMF parameters employed by Ko et al. (64) were consistent with those of Alfredo et al. (90, 91), which mimic mitochondrial-driven regenerative and metabolic pathways activated by exercise, primarily aimed at muscle tissue repair. Notably, this therapy can enhance mitochondrial bioenergetics in muscle and mitigate systemic lipotoxicity (92). Although the parameter of the PEMF therapy is supported by *in vitro* studies showing enhanced muscle cell proliferation (91), as well as findings from animal models (93) and clinical trials in humans (92, 94, 95), the effectiveness of PEMF therapy within this frequency and intensity parameter in treating symptoms among individuals with tendinopathy remains uncertain and warrants further investigation.

However, the lack of a between-group difference in the Ko et al. (64) study may be due to masking effects from the prescribed eccentric exercises. Previous systematic reviews have demonstrated that eccentric exercise significantly improves both pain and function in patients with tendinopathy, outperforming other exercise modalities and passive approaches such as “wait-and-see” strategies (31, 96). This is further supported by previous evidence suggesting that combining PEMF therapy with rehabilitative exercise yields no additional benefits, as rehabilitative exercise alone may partially influence both pain and functional outcomes (97–99). The mechanism of eccentric exercise involves muscle contraction during lengthening. These contractions generate higher forces than concentric or isometric contractions and are more energy-efficient (100). This contributes to improved mechanical properties and increased collagen synthesis through enhanced blood flow, oxygen uptake, and metabolism, as well as stimulation of both collagen degradation and regeneration (64). Conversely, the study by Gerdesmeyer et al. (63) utilises heel cushions as the control treatment, which have limited evidence of its effectiveness in the management of AT as compared to eccentric exercise. Hence, the reported clinical improvements are likely attributable to PEMF therapy rather than to the heel cushion intervention itself. However, the lack of blinding of assessors, therapists, and participants introduces a high risk of bias, and thus, the findings should be interpreted with caution.

With regard to the effects of PEMF therapy on self-reported physical function, our systematic review identified only one study conducted by Öztürk et al. (65) that demonstrated a significant improvement in self-reported physical functions in the PEMF therapy. Specifically, the combination of PEMF therapy and ESWT resulted in an average improvement of 35 points the FFI activity limitation subscale compared to ESWT alone (*p* < 0.001). Notably, the improvement in self-reported physical functions was aligned with the highest reduction in pain score observed across the included studies*.* This finding from the review supports the hypothesis that self-reported measures of physical functioning is closely associated to the pain intensity (101). Pain may act as a central mediator influencing functional capacity, as patients experiencing increasing pain demonstrate avoidance behaviours leading to reduced muscle strength, impaired balance, decreased range of motion, all of which can affect physical activities, contributing to muscle wasting and function decline (102, 103). Thus, the reduction in pain symptoms may lead to perceived functional improvement through peripheral and central mechanisms, including the changes in neuromuscular activation and inflammatory modulation.

Conversely, neither Ko et al. (64) nor Gerdesmeyer et al. (63) reported significant improvements in physical function following the intervention. This outcome may reflect the complex interplay of factors influencing functional capacity, beyond pain alone. Biophysical limitations, psychological factors, and comorbidities are also likely to contribute to participants' self-perceived functional ability (48). Future studies should include objective functional assessments, such as muscle strength measurements or standardised performance-based tests, to more accurately evaluate treatment effects. Indeed, evidence suggests that clinically meaningful improvements in physical function generally require substantial increases in muscle strength—estimated at approximately 30%–40% (104). It is therefore possible that the PEMF therapy protocols used in the included studies did not reach a sufficient physiological threshold to produce a measurable effect on self-reported functional outcomes (95). Nonetheless, our review includes only three randomised controlled trials on two high-intensity PEMF therapies and one study on low-intensity PEMF therapy, which prevents any clear recommendation regarding the use of PEMF therapy at this stage. Therefore, additional randomised controlled trials with standardised PEMF therapy protocols and long-term follow-ups are needed to determine whether high- or low-intensity PEMF therapy may offer beneficial effects for individuals with foot and ankle soft-tissue injuries.

### 4.1 Reports of adverse events

Pennington et al. (62), Ko et al. (64), and Oztürk et al. (65) reported no adverse effects, and the treatment was well-tolerated by the patient. On the contrary, minor temporary redness was reported by Gerdesmeyer et al. (63). Nonetheless, no serious device-related adverse events were reported, reinforcing the safety profile of the PEMF therapy. Future studies may include standardised and comprehensive adverse event reporting to ensure more accurate assessments of treatment safety.

### 4.2 Limitations

This systematic review presents several limitations that warrant careful interpretation. A primary limitation is due to individual limitations of the included studies, mainly due to the small smaple size (*n* = 243) coupled with high heterogeneity of the PEMF therapy protocols, controlled therapies, and treatment regimen, including frequency, intensity, session duration, total number of sessions, and overall treatment duration. Such variability may hinder the establishment of a standardised therapeutic protocol and interfere with the interpretation of treatment efficacy across different clinical contexts. Moreover, the heterogeneity of patient populations across studies limits the generalizability of the findings. The effectiveness of PEMF therapy demonstrated in one subgroup (for e.g., Achilles tendinopathy) may not be extrapolated to other disease groups. Future investigations may focus on a more homogeneous population to evaluate the effectiveness of the PEMF therapy.

Another methodological concern is the inconsistency in outcome measures across studies, which hinders meaningful comparisons or meta-analysis. Standardisation of outcome assessments and dosage protocols should be established across the various clinical conditions in future studies. For instance, incorporating both subjective and objective measures should be a priority for future trials. In particular, the reliance on self-reported physical function outcomes in three of the four studies raises concerns about potential recall bias and subjective variability. Objective assessments, such as strength testing, range of motion, and balance evaluation, should be integrated to enhance the reliability of functional outcomes. Additionally, none of the included studies were controlled for medication use, particularly NSAIDs, which may influence pain perception and inflammatory responses. This may confound the effects of PEMF therapy. Future RCTs should rigorously control for pharmacological interventions to isolate the actual therapeutic effects of PEMF therapy.

Long-term efficacy and safety remained unclear due to the limited follow-up duration, with the longest follow-up extending only 12 weeks. This restricts our understanding of the sustained benefits or potential delayed adverse effects of PEMF therapy. Well-designed, long-term studies are needed to address this gap.

Furthermore, one study demonstrated significant methodological shortcomings, including incomplete blinding, inadequate randomisation, and failure to document comorbidities, all of which may introduce bias and compromise internal validity. The review's search strategy also excluded grey literature and non-English publications, potentially omitting relevant evidence and introducing publication bias.

In summary, heterogeneity across study populations, PEMF parameters, outcome measures, and methodological quality collectively limits the robustness and generalisability of current findings. Future research should prioritise rigorous, large-scale RCTs with standardised intervention protocols, comprehensive outcome assessments, and extended follow-up periods to better establish the long-term safety and effectiveness of PEMF therapy in specific musculoskeletal conditions.

## 5 Conclusion

The systematic review suggests that high intensity, low frequency PEMF therapy may offer additional pain relief for musculoskeletal soft-tissue injuries, but the evidence regarding PEMF therapy in improving self-reported physical function among foot and ankle soft-tissue injuries remained uncertain. Nevertheless, the evidence should be interpreted with caution due to high heterogeneity and a possible risk of bias, resulting in low-quality evidence. Future research should include objective measures of physical function alongside self-reported outcomes to improve the methodological robustness and generalisability of findings.

## Data Availability

The original contributions presented in the study are included in the article/Supplementary Material, further inquiries can be directed to the corresponding author.
