# Supplementary material for: The effects of pulsed electromagnetic field therapy on pain and physical functions in patients with soft tissue injuries: a systematic review of randomised controlled trials
Source: Front Sports Act Living. 2026 Feb 5;8:1694944. doi: 10.3389/fspor.2026.1694944 (PMC12916110; doi:10.3389/fspor.2026.1694944)
Supplement: Supplementary file 1 [file Table1.docx]

Supplementary Material

Supplementary Data: Appendix of the mean difference between the control and intervention groups in various foot and ankle pathologies (foot functions)

| **Reference** | **Scale** | **Control/Comparator** |  | **Experimental** |  |  | **Improvement between control and experimental** |
| --- | --- | --- | --- | --- | --- | --- | --- |
|  |  | Mean (SD) at final follow-up | MD from baseline | Mean (SD) at final follow-up | MD from baseline | MD between both groups |  |
| **Calcaneal Spurs** | | | | | | |  |
| Ozturk et al., 2023 | Foot Function Index (Functional limitation) | 40 (27) | -5 | 5 (5.75) | -40 | 35 | Significant improvement between two groups. |
| **Achilles Tendinopathy** | | | | | | |  |
| Gerdesmeyer et al., 2017 | Role-Maudsley | 2.92 (0.78) | -0.76 | 2.57 (0.92) | -1.04 | 0.35 | No significant improvement between the two groups. |
| Ko et al., 2024 | Victorian-Institute of Sport Assessment-Achilles | 66.75 (23.07) | 11.65 | 71.70 (18.01) | 13.85 | 4.95 | No significant improvement between the two groups. |
